# Sex Differences in Social Adaptive Function in Autism Spectrum Disorder and Attention-Deficit Hyperactivity Disorder

**DOI:** 10.3389/fpsyt.2019.00607

**Published:** 2019-09-12

**Authors:** Tania Mahendiran, Annie Dupuis, Jennifer Crosbie, Stelios Georgiades, Elizabeth Kelley, Xudong Liu, Robert Nicolson, Russell Schachar, Evdokia Anagnostou, Jessica Brian

**Affiliations:** ^1^Faculty of Medicine, Institute of Medical Science, University of Toronto, Toronto, ON, Canada; ^2^Autism Research Centre, Bloorview Research Institute, Toronto, ON, Canada; ^3^University of Toronto, Dalla Lana School of Public Health, Toronto, ON, Canada; ^4^Department of Psychiatry, The Hospital for Sick Children, Toronto, ON, Canada; ^5^Department of Psychiatry and Behavioural Neurosciences, McMaster University, Hamilton, ON, Canada; ^6^Department of Psychology, Queen’s University, Kingston, ON, Canada; ^7^Department of Psychiatry, Queen’s University, Kingston, ON, Canada; ^8^Department of Psychiatry, Western University and Children’s Health Research Institute, London, ON, Canada; ^9^Department of Pediatrics, University of Toronto, Toronto, ON, Canada

**Keywords:** autism spectrum disorder, sex differences, attention-deficit hyperactivity disorder, neurodevelopmental disorders, social-communication behaviours

## Abstract

**Background:** Social-communication difficulties, a hallmark of ASD, autism spectrum disorder (ASD) are often observed in attention – deficit/ hyperactivity disorder (ADHD), although are not part of its diagnostic criteria. Despite sex differences in the prevalence of ASD and ADHD, research examining how sex differences manifest in social and communication functions in these disorders remains limited, and findings are mixed. This study investigated potential sex differences with age in social adaptive function across these disorders, relative to controls.

**Method:** One hundred fifteen youth with ASD, 172 youth with ADHD, and 63 typically developing controls (age range 7–13 years, 75% males) were recruited from the Province of Ontario Neurodevelopmental Disorder (POND) Network. Social adaptive function was assessed using the Adaptive Behavior Assessment System-Second Edition (ABAS-II). The proportions of adaptive behaviors present in each skill area were analyzed as a binomial outcome using logistic regression, controlling for age, and testing for an age-by-sex interaction. In an exploratory analysis, we examined the impact of controlling for core symptom severity on the sex effect.

**Results:** Significant sex-by-age interactions were seen within ASD in the communication (p = 0.005), leisure (p = 0.003), and social skill areas (p < 0.0001). In all three areas, lower scores (indicating poorer function) were found in females compared to males at older ages despite females performing better at younger ages. There were significant differences in the sex-by-age interactions in the social and leisure domains between those with ASD and typically developing controls, with typically developing females showing better scores at older, compared to younger, ages. There were also significant differences in the sex-by-age interactions between ASD and ADHD on the social and leisure domains, as females with ADHD consistently scored higher on social skills than males across all ages, unlike those with ASD. Sex differences across age in the social domains for ADHD were similar to those in the typically developing group.

**Conclusion:** Sex differences in social and communication skill areas were observed between ASD and ADHD, and typically developing controls, with females with ASD performing worse than males at older ages, despite an earlier advantage. These findings reinforce the need to take a developmental approach to understanding sex differences which may have diagnostic, prognostic, and treatment implications.

## Introduction

Autism spectrum disorder (ASD) and attention-deficit/hyperactivity disorder (ADHD) are common neurodevelopmental disorders. Autism spectrum disorder is characterized by deficits in social communication, as well as restricted and repetitive behaviors and interests (1). Attention-deficit/hyperactivity disorder (ADHD) is characterized by inattention, hyperactivity, and impulsivity that interfere with function (1, 2). According to both Canadian and US surveillance studies, the prevalence of ASD is about 1.5% [National Autism Spectrum Disorder Surveillance System Report (3)] (4). The prevalence of ADHD is estimated at 5–7% (5). Even though the Diagnostic and Statistical Manual of Mental Disorders, 5^th^ edition (DSM-5) (1) diagnostic criteria for these disorders appear to show little symptom overlap, the two disorders frequently co-occur. The prevalence of ADHD in individuals with ASD has been reported in the range of 30 to 80%, while ASD is estimated to occur in 20% to 50% of individuals with ADHD (6–8). Moreover, overlapping behavioral traits have been reported in both youth with ASD and those with ADHD, including inattention, hyperactivity, social impairment, and repetitive behaviors (9–13).

Both ASD and ADHD are characterized by a male predominance (14, 15). In epidemiological studies, the male to female ratio in ASD ranges from 1.33:1 to 16:1 (16, 17). The most recent male to female ratio in ASD was reported to be 4:1 (4). The sex ratio varies by cognitive ability, with higher ratios (10:1) in individuals with higher cognitive abilities (IQ) but lower ratios (2:1) in individuals with comorbid intellectual disability (16, 18). In children with ADHD, male to female ratio estimates range from 10:1 to 3:1 in clinical and community samples, respectively (14).

In the context of prominent sex differences in the prevalence of ASD and ADHD, it is important to understand how such sex differences may interact with specific symptom domains. For example, males with ASD have been reported to exhibit more repetitive behaviors than females (19–21) while females with ADHD have been found to have less inattention, hyperactivity/impulsivity, and fewer total ADHD symptoms than males with ADHD (22). A better understanding of as yet underexplored sex differences in symptom domains across ASD and ADHD may help us elucidate the biological underpinnings of these disorders, characterize possible sex-specific profiles, and potentially influence the development of treatments.

Social-communication difficulties, a hallmark of ASD, are often observed in ADHD although not part of its diagnostic criteria. Interpersonal difficulties, peer rejection, and social problems are prominent in ADHD (23, 24). Greater impairments in peer relations (25) and poor friendship quality and stability (26, 27) have been reported. Children with ADHD have few reciprocated friendships, are rated by peers as less-preferred socially, (25) and are more likely to be disliked by their peers compared to typically developing children (28, 29). A systematic review by Kok et al. (30) on social skills in children with ADHD reported social deficits in females with ADHD compared to typically developing female peers. Specifically, females with ADHD experienced less positive peer interactions, and lower rates of friendship participation and stability compared to same-aged typically developing females. Martel et al. (31) reported significant deficits on the social problems domain of the Child Behavior Checklist in children with ADHD compared to controls. Studies using both measures of autistic traits and more global measures of social deficits continue to identify social impairments in ADHD (31–35). Using an autism criteria checklist (32), children with ADHD presented with deficits in the desire to interact with others had problems with non-verbal communication and poor eye contact and had difficulty forming relationships.

Evidence of sex differences in social-communicative abilities is mixed in both ASD and ADHD (5, 36, 37). In the case of ASD, some studies have reported that males with ASD had more social-communication deficits than females (38–42), while other studies have found no sex differences (21, 43–47), and yet another few studies have reported more social difficulties in post-pubescent girls than boys with ASD (48, 49). In the case of ADHD, most of the research has focused on males (50), making it difficult to characterize the role of sex differences. Studies of sex differences in peer functioning among children with ADHD are few and have yielded contradictory results (5, 36). Studies of community samples have shown that females were more likely than males with ADHD to be rejected and disliked by peers; however, studies of clinical samples reported that males had more parent-reported peer problems than females (51, 52), and yet others have found no differences (24, 53).

These inconsistencies across studies could be the result of power issues stemming from small samples of females, variability in measures used, as well as possible changes in symptoms across development. Of note, there are limited studies examining sex differences across age. To date, McLennan et al. (49) study is the only longitudinal study that has explored sex-specific trajectories in ASD symptoms, where females were found to be more impaired than males in ratings of social function and reciprocal friendships after age 10.

In typically developing children, quantitative and qualitative research has suggested that females engage in more prosocial behavior (54), express greater concern regarding others’ feelings (55, 56), and spend more time in dyadic interactions than males (57, 58). Also, females usually have tighter and more intimate social networks and peer relationships than males that involve higher peer attachment (58). Moreover, formation of intimate social groups and group affiliations increases more during adolescence for females than for males (59). Age effects have also been noted, with more improvements in associative play at age 3–4, cooperative play at 4–5 and social interactions with peers at ages 5–6 in females than in males (57).

In summary, research in sex differences in social-communication function in ASD and ADHD is inconclusive. Inconsistencies may be due to variations in methodology, power issues due to smaller female samples, possible changes in skills, and symptoms across development, or may reflect a real lack of robust sex differences. Moreover, most ASD and ADHD research in this area has not included a typically developing control group, making it difficult to determine whether the observed male to female differences are a reflection of typical sex effects across development.

The aim of the current study is to understand the pattern of potential sex differences in social adaptive function in ASD and ADHD and compare them to typically developing controls.

Note that this is a cross-sectional study, and as such any age-by-sex interactions are only suggestive of changes with age. For ease of communication, we occasionally use terms such as “increase,” “improve,” or “decline,” but acknowledge that our findings are not based on longitudinal data.

## Method

### Participants

The present study included children between the ages 7 and 13 years with diagnosis of ASD or ADHD, and typically developing (TD) controls. The data were accessed from the Province of Ontario Neurodevelopmental Disorders (POND) Network database, a research network across five Ontario universities and hospitals (Holland Bloorview Kids Rehabilitation Hospital, the Hospital for Sick Children; McMaster University and the Offord Centre; the Lawson Health Research Institute; and Queen’s University). Typically developing controls were volunteers from the community with no first degree relative with a neurodevelopmental disorder. This study was specifically reviewed and approved by an ethics committee. Written and informed parental consent was obtained for all participants under the age of 16.

Measures: Diagnosis of ASD was supported by the Autism Diagnostic Observation Schedule-2 (ADOS-2) (60), and the Autism Diagnostic Interview-Revised (ADI-R) (61). Diagnosis of ADHD was confirmed using the parent interview for child symptoms (PICS) (62). Participants’ parents completed the Adaptive Behavior Assessment System-Second Edition (ABAS-II) parent-report measure (63). Intellectual ability (IQ) was estimated using a Wechsler scale (Wechsler Abbreviated Scale of Intelligence (WASI-I or-II) (64, 65), Wechsler Intelligence Scale for Children-4^th^ edition (WISC-IV) (66), or the Stanford Binet Intelligence Scales (67), when a Wechsler scale was not appropriate.

### Adaptive Behavior Assessment System-Second Edition (ABAS-II) (63)

The ABAS-II parent-report measure was used in the present study to assess social and communication functions for children diagnosed with ASD, ADHD, and typically developing controls. This parent-report measure assesses an individual’s daily adaptive functioning. The measure consists of 10 skill areas: communication, community use, functional academics, home living, health and safety, leisure, self-care, self-direction, social, and work skills. Parents or guardians were asked to assess how often their child engages in a particular activity using a 4-item Likert scale (0—is not able, 1—never when needed, 2—sometimes when needed, 3—always when needed). The present study examined scores on the communication, leisure, and the social skill areas of the ABAS-II questionnaire. We selected the social, communication, and leisure areas, as they are arguably the most relevant to a child’s ability to adapt to broadly conceptualized social demands of day–day life. To capture this concept in a way that is not overly cumbersome to the reader, we used the term “social adaptive function” throughout. The ABAS-II communication skill area consists of 24 items and assesses pragmatic language and listening skills. The leisure skill area consists of 22 items that assesses the individual’s ability and frequency to plan and organize leisure and/or recreational activities, while the social skill area consists of 23 items assessing peer interaction and ability to form friendships. The test–retest reliability coefficients of the adaptive domains range from 0.80 and 0.90s. The inter-rater reliability coefficients of the GAC (General Adaptive Composite—which is derived from the sum of scaled scores from the 10 skill areas and is thought to represent a comprehensive estimate of an individual’s overall adaptive functioning) are 0.91 (ages 10–21), and the average corrected reliability coefficients of the skill areas of each performance level ranges from 0.78 to 0.98 (63). The ABAS-II is a measure, with norms from the general population, which assesses social and communication adaptive functions across a broad range, and is not designed to assess social-communication deficits that are specific to ASD or to any other specific disorder. As the present study includes a cross-disorder analysis, we selected this measure to ensure that the same construct is measured across disorders. Moreover, the ABAS-II measures adaptive or “real-world” social and communication functions (rather than skills or deficits), which provides an index of an individual’s competency in everyday contexts.

### Measures Used in Exploratory Analyses

To assess whether trait severity (estimated by number of symptoms) is associated with observed sex differences in social adaptive function, the following parent-reported questionnaires were available: the Social-Communication Questionnaire (SCQ; 68) to estimate social and communication deficits (items testing for repetitive behavior on the SCQ were removed from the analysis), Strength and Weakness of ADHD Symptoms and Normal Behavior Rating Scale (SWAN) (69) to estimate hyperactivity and impulsivity symptoms, and the Repetitive Behavior Scale-Revised (RBSR) to estimate repetitive behaviors.

### Analytic Plan

**Figure 1:**
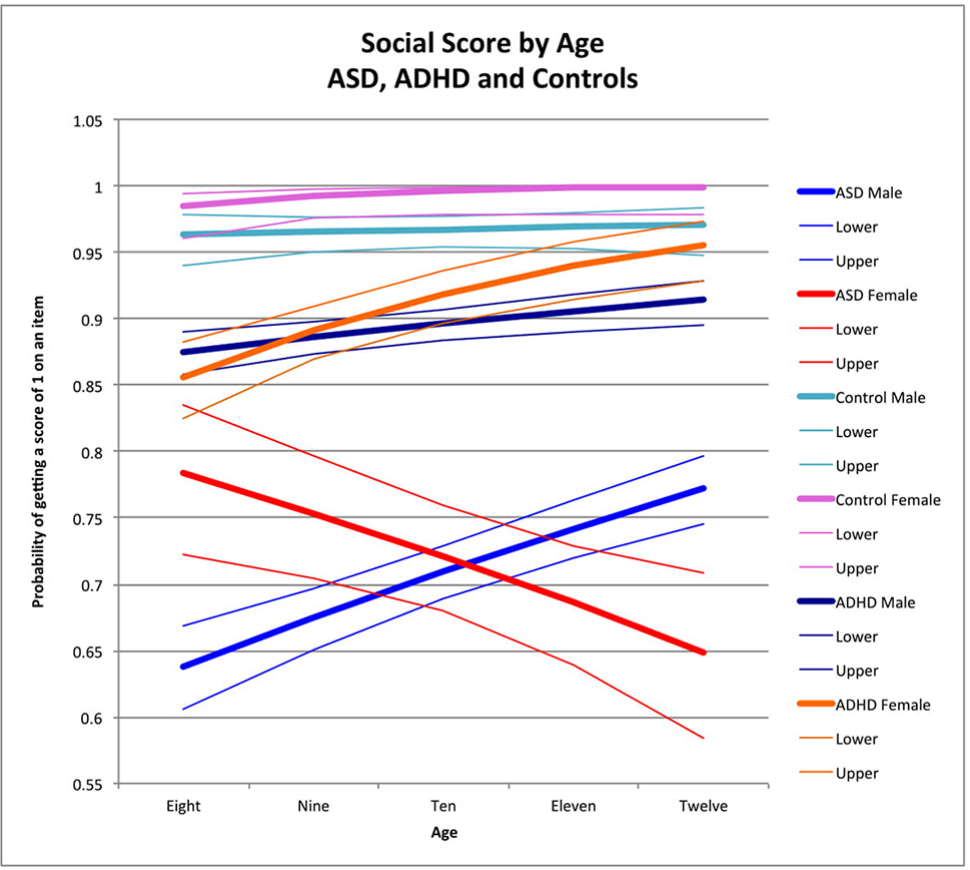
Social score by age: ASD, ADHD, and controls. This graph depicts the probability of obtaining a positive score of 1 on an individual item in the social skill area (indicating skill is present) across ages in ASD (where males are in blue while females are in red), in ADHD (where males are in dark blue and females are in orange), and in typically developing controls (where males are in light blue and females are in pink). Dx, diagnosis; ASD, Autism Spectrum Disorder; ADHD, Attention Deficit Hyperactivity Disorder.

**Figure 2:**
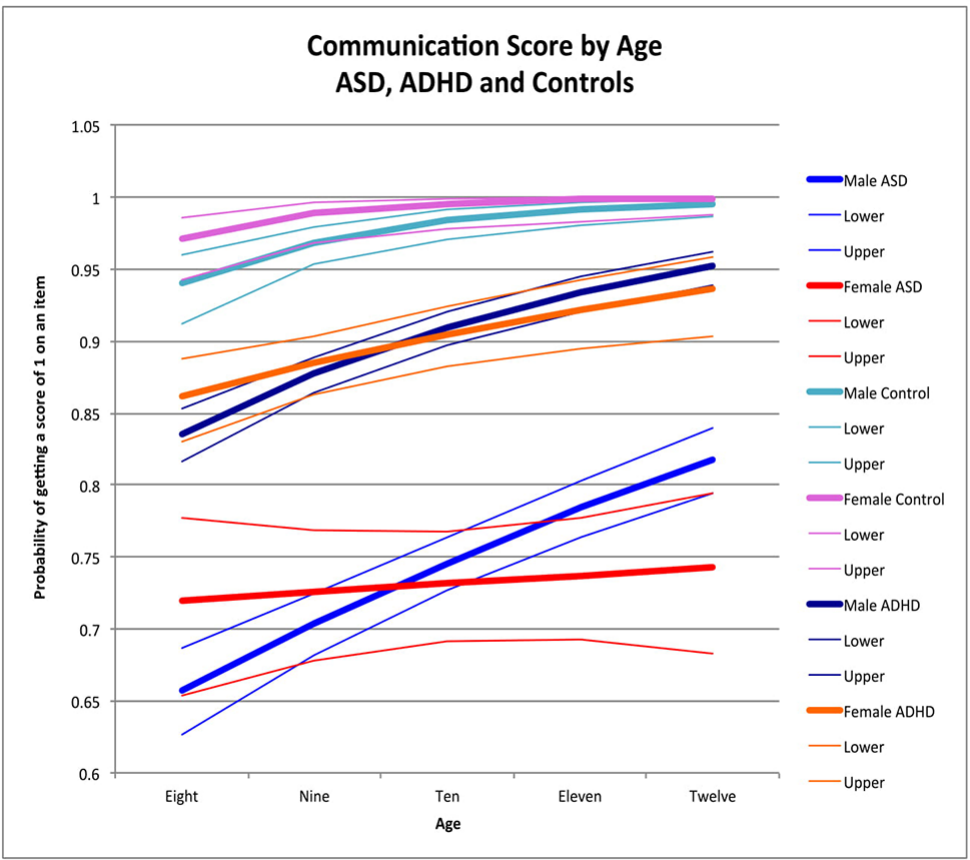
Communication score by age: ASD, ADHD, and controls. This graph depicts the probability of obtaining a positive score of 1 on an individual item in the communication skill area (indicating skill is present) across ages in ASD (where males are in blue while females are in red), ADHD (where males are dark blue while females are orange), and in typically developing controls (where males are in light blue and females are in pink). Dx, diagnosis; ASD, Autism Spectrum Disorder; ADHD, Attention Deficit Hyperactivity Disorder.

**Figure 3:**
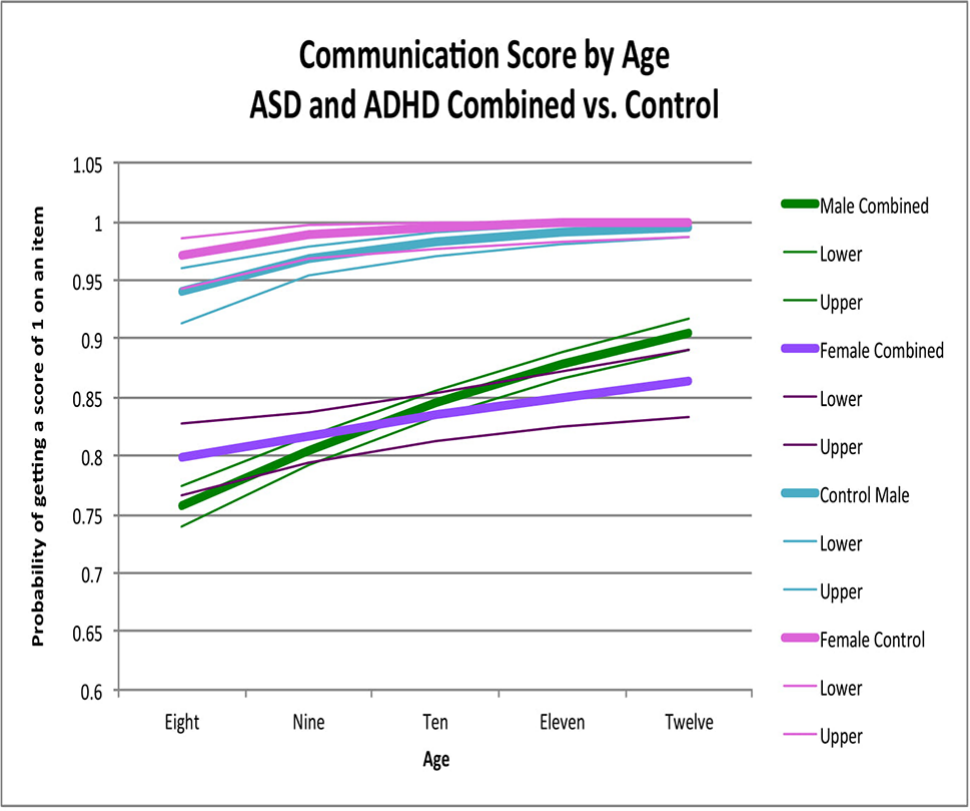
Communication score by age: ASD and ADHD combine versus control. This graph depicts the probability of obtaining a positive score of 1 on an individual item in the communication skill area (indicating skill is present) across ages in ASD and ADHD combined model (where males are in green while females are in purple) and in typically developing controls (where males are in light blue and females are in pink). Dx, diagnosis; ASD, Autism Spectrum Disorder; ADHD, Attention Deficit Hyperactivity Disorder.

**Figure 4:**
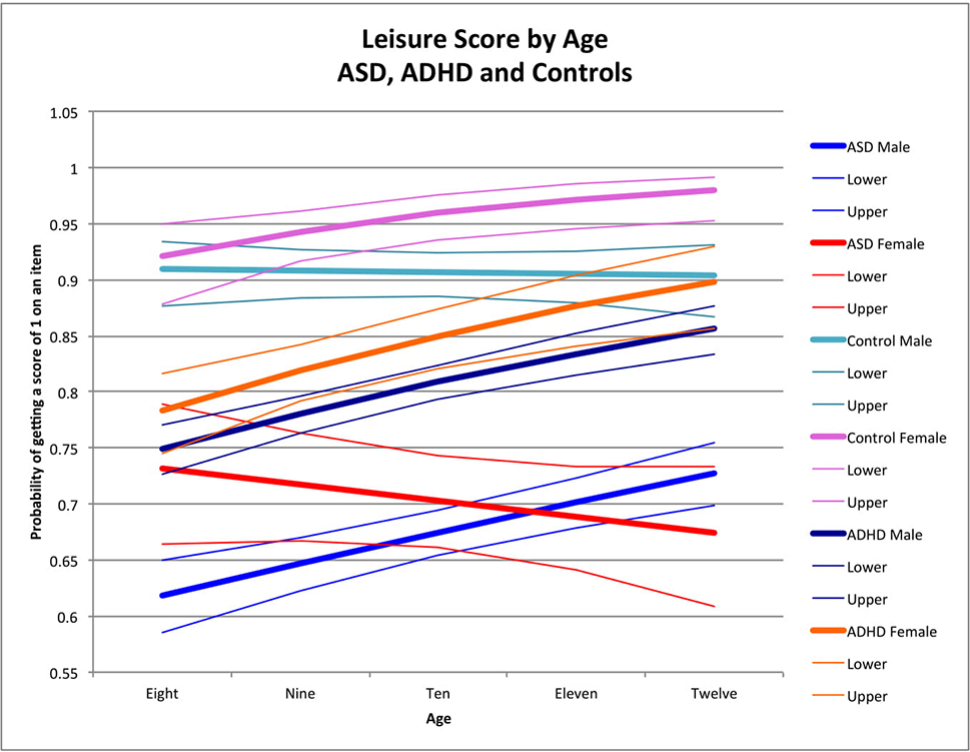
Leisure score by age: ASD, ADHD, and controls. This graph depicts the probability of obtaining a positive score of 1 on an individual item in the leisure skill area (indicating skill is present) across ages in ASD (where males are in blue while females are in red), ADHD (where males are dark blue while females are orange), and in typically developing controls (where males are in light blue and females are in pink). Dx, diagnosis; ASD, Autism Spectrum Disorder; ADHD, Attention Deficit Hyperactivity Disorder.

ABAS-II item scores were transformed into dichotomous variables. To accomplish this, scores of 0 or 1 were converted to “0” (corresponding to the absence of a skill), and scores of 2 or 3 were converted to “1” (i.e., the presence of that skill). We then analyzed the proportion of behaviors present in each skill area as a binomial outcome using logistic regression, controlling for age and sex. The advantage of dichotomizing reduces the variability due to parental expectation of appropriate frequency of the skills, which increases our confidence that a particular skill is present or absent. All analyses were performed using SAS 9.4 (2002–2010 by SAS Institute Inc., Cary NC, USA). We first examined the age effect across males and females within each group (ASD, ADHD, and controls) by including an age × sex interaction in the model. Where the interaction was significant, we estimated the sex effect across a range of integer ages to facilitate interpretation of the interaction effect. Where the interaction was not significant, we reported the overall sex effect. We then used the estimated coefficients from the final models to predict the proportion of symptoms at ages 8, 9, 10, 11, and 12 to provide scenarios for graphical representation to help interpret the impact of age-by-gender interaction terms and, when the interaction was not significant, to show the effect of age across both males and females. The graphs (**Figures 1**–**4**) display the probability of getting a score of 1 on any individual item, which also corresponds to the expected proportion of adaptive behaviors present. For example, seen in **Figure 1**, boys scored positively (i.e., positive score of 1, indicating presence of adaptive behavior) on 64% of the items in the social skill area whereas girls scored positively on 78% of the items.

We then compared diagnostic groups to each other and to controls to determine if sex effects differed between them. Where the age-by-sex effect was significant within one or both diagnoses (dx), a three-way interaction was included in the combined model to test for differences in the age-by-sex effect between the different groups. Where this interaction was significant, the sex effect between diagnoses was then evaluated at different points across the age range to characterize the impact of the three-way interaction. Where the three-way interaction was not significant, the overall sex effect was evaluated using a sex-by-diagnosis (dx) interaction.

If no significant three-way interaction and age-by-sex-by-diagnosis (dx) interaction was found between ADHD and ASD, then both groups were combined into one model for the purpose of comparing to controls.

### Exploratory Analysis

To determine whether symptom severity, as approximated by symptom count, influenced sex differences in ASD and ADHD, items of the symptom-/trait-based measures that assess social and communication deficits, inattention and hyperactivity/impulsivity, and repetitive behaviors (SCQ, SWAN, RBSR) were then added to the models. Scores on the SWAN and RBSR were dichotomized to correspond to presence/absence of a symptom, to be comparable to the SCQ. We explored the proportion of variability in the sex effect within and across disorders that was accounted for by the number of symptoms, by inspecting sex-by-age-by-diagnosis interactions in each domain before and after controlling for symptom counts. We examined the effect of adding trait measures to the ASD and ADHD models separately, both visually and by looking at the change in significance level for the sex-by-age interaction where significant. As there are no objective criteria for characterizing the magnitude of the change in sex-by-age effects, we only report these effects qualitatively with a focus on overall trends and not on individual changes.

## Results

### Study Sample

A total of 350 children were included in the analyses. Sample information and demographics are reported in **Table 1**. The overall age range was 7–13 years, and 75% of the sample was male. No significant differences were noted between males and females in IQ within ASD, ADHD, and controls.

**Table 1:** Sample characteristics and information.

	ASD			ADHD			Controls		
Males (%)	93 (81)			128 (74)			41 (65)		
	Mean (SD)								
n	115			172			63		
Comorbid ADHD or ASD	6			2					
	Male	Female	T-test	Male	Female	T-test	Male	Female	T-test
**Age**	10.1 (1.8)	10.1 (1.7)	t=0.00 p>0.9	9.6 (1.7)	9.3 (1.5)	t=1.03 p=0.3	9.9 (1.6)	9.9 (1.8)	t=0.00 p>0.9
**IQ**	85.6 (24.1)	88.3 (20.9)	t=0.48 p=0.6	102.4 (16.2)	98.3 (15.6)	t=1.5 p=0.1	108.9 (12.3)	113.2 (10.3)	t=1.40 p=0.2
**ABAS communication score**	4.7 (3.1)	4.8 (3.5)	t=0.13 p=0.9	7.6 (2.9)	8.7 (3.2)	t=2.1 p=0.04	10.9 (2.3)	12.2 (2.1)	t=2.20 p=0.03
**ABAS leisure score**	5.7 (2.8)	6.0 (3.0)	t=0.45 p=0.7	7.8 (2.9)	9.4 (3.0)	t=3.12 p=0.002	10.7 (2.5)	13.0 (1.9)	t=3.77 p=0.0004
**ABAS social score**	3.4 (3.0)	4.0 (3.8)	t=0.80 p=0.4	6.7 (3.5)	8.4 (3.5)	t=2.77 p=0.006	9.8 (3.1)	12.3 (1.8)	t=3.47 p=0.001
**SWAN scores**	8.5 (5.2)	7.6 (5.3)	t=0.72 p=0.5	10.6 (5.0)	9.4 (5.0)	t=1.37 p=0.2	0.2 (0.6)	0.4 (1.7)	t=0.68 p=0.5
**SCQ scores**	18.1 (7.1)	17.0 (7.3)	t=0.65 p=0.5	7.0 (5.0)	6.2 (4.8)	t=0.92 p=0.4	3.0 (2.3)	1.8 (1.4)	t=2.23 p=0.03
**RBSR scores**	8.9 (8.0)	9.2 (8.7)	t=0.16 p=0.9	3.5 (4.9)	3.8 (5.5)	t=0.34 p=0.7	0.1 (0.5)	0.3 (0.9)	t=1.14 p=0.3
**ABAS GAC**	66.9 (15.2)	66.3 (16.2)	t=0.16 p=0.9	80.2 (14.4)	84.0 (14.2)	t=1.51 p=0.1	96.6 (11.9)	108.6 (11.6)	t=3.84 p=0.0003

SWAN, SCQ, and RBSR scores are totals after dichotomizing the individual item scores into 0 (for absent) and 1 (for present). ABAS-II GAC scores are standardized total scores which summarizes performance across all skill areas on the ABAS-II, except for Work.

SD, standard deviation; ABAS, Adaptive Behavior Assessment System-Second Edition; SCQ, Social Communication Questionnaire; SWAN, Strengths and Weaknesses of ADHD symptoms and Normal Behaviour Rating Scale; RBSR, Repetitive Behavior Scale-Revised; GAC, General Adaptive Composite.

### Main Group Differences

Overall, typically developing children outperformed children with ADHD, and both groups outperformed children with ASD across all three domains on the ABAS-II (raw scores—communication skill area F = 100.8, p < 0.0001; leisure skill area F = 80.1, p < 0.0001; social skill area F = 94.6, p < 0.0001) (see **Table 2** for the pairwise comparisons). Females outperformed males on the communication, leisure, and social skill areas in both the ADHD and control groups but not in the ASD group (please see **Table 1** for the demographic information as well as **Supplemental Tables 4, 5**, and **6** for the mean scores of male and female participants across age).

**Table 2:** Pairwise comparisons.

Communication				
	Mean difference	Standard error	Confidence interval	Significance
Control vs. ASD	18.83*	1.38	15.5–22.1	p<0.0001
Control vs. ADHD	8.51*	1.30	5.4–11.6	p<0.0001
ADHD vs. ASD	10.32*	1.06	7.8–12.9	p<0.0001
Leisure				
	Mean difference	Standard error	Confidence interval	Significance
Control vs. ASD	16.31*	1.30	15.8–22.8	p<0.0001
Control vs. ADHD	9.09*	1.22	5.1–11.8	p<0.0001
ADHD vs. ASD	7.22*	1.00	8.1–13.5	p<0.0001
Social				
	Mean difference	Standard error	Confidence interval	Significance
Control vs. ASD	19.27*	1.46	13.2–19.4	p<0.0001
Control vs. ADHD	8.45*	1.38	6.1–12.03	p<0.0001
ADHD vs. ASD	10.815*	1.13	4.8–9.6	p<0.0001

Based on estimated marginal means.

*The mean difference is significant at the 0.05 level, adjustment for multiple comparisons: Bonferroni.

### Sex Differences in Social, Communication, and Leisure Skills in ASD, ADHD, and Controls

#### Social Skill Area

Older children obtained higher social scores than younger children among males with ASD (male OR for age = 1.18 p < 0.0001) (**Figure 1**; note that y-axis depicts the probability of getting a score of 1 on an individual item) and children with ADHD across both sexes (female OR for age = 1.11, *p* = 0.002; male OR for age = 1.35, *p* < 0.0001) (**Figure 1**). In ADHD, there was a significant difference in the effect of age between the two sexes in ADHD (χ^2^ = 5.47, *t*0.02); specifically, both sexes had better performance across age, but the magnitude was greater in females (**Table 3**; **Figure 1**). There was a significant negative effect of age in females with ASD (female OR for age = 0.85, *p* < 0.0001) resulting in a significant difference between males and females with ASD (χ^2^ = 1.18, *p* < 0.0001) (**Figure 1**). There was no significant effect of age in male and female controls (male OR for age = 1.6, *p* = 0.6; female OR for age = 2.1, *p* = 0.07). Sex-by-age interactions between participants with ASD and controls reached statistical significance (sex-by-age-by-diagnosis interaction: χ^2^ = 5.37, *p* = 0.02).

**Table 3:** Age effects and sex by age interactions for ASD, ADHD, and controls.

	ASD	ADHD	Control
Social skill area	*OR (95% CL)* *p value*	*OR (95% CL)* *p value*	*OR (95% CL)* *p value*
Male age effect	1.18 (1.12; 1.24) <.0001	1.11 (1.04; 1.20) 0.002	1.06 (0.85; 1.32) 0.6
Female age effect	0.85 (0.75; 0.95) 0.0001	1.35 (1.17; 1.57) 0.0001	2.12 (0.93; 4.84) 0.07
Sex × age interaction χ^2^; p value	26.03; <.0001	5.47; 0.02	2.50; 0.1
Diagnosis x sex × age interaction ADHD vs. ASD χ^2^; p value		24.94; 0.0001	
Diagnosis x sex × age interaction ASD vs. control χ^2^; p value		5.37; 0.02	
Diagnosis x sex × age interaction ADHD vs. control χ^2^; p value		1.25; 0.3	
Leisure skill area	*OR (95% CL)* *p value*	*OR (95% CL)* *p value*	*OR (95% CL)* *p value*
Male age effect	1.13 (1.08; 1.19) <0.0001	1.19 (1.13; 1.26) < 0.0001	0.98 (0.86; 1.13) 0.8
Female age effect	0.93 (0.83; 1.05) 0.2	1.25 (1.11; 1.41) 0.0002	1.43 (1.11; 1.85) 0.006
Sex × age interaction χ^2^; p value	8.97, 0.003	0.58, 0.5	6.35, 0.01
Diagnosis x sex × age interaction ADHD vs. ASD χ^2^; p value		6.91; 0.009	
Diagnosis x sex × age interaction ASD vs. control χ^2^; p value		12.29; 0.0005	
Diagnosis x sex × age interaction ADHD vs. control χ^2^; p value		3.93; 0.05	
Communication skill area	*OR (95% CL)* *p value*	*OR (95% CL)* *p value*	*OR (95% CL)* *p value*
Male age effect	1.24 (1.17; 1.30) < 0.0001	1.41 (1.30; 1.52) < 0.0001	1.96 (1.42; 2.69) < 0.0001
Female age effect	1.03 (0.92; 1.16) 0.6	1.24 (1.08; 1.42) 0.002	2.63 (1.29; 5.37) 0.5
Sex × age interaction χ^2^; p value	8.07, 0.005	2.57, 0.1	0.56, 0.5
Diagnosis x sex × age interaction ADHD vs. ASD χ^2^; p value		0.31; 0.5786	
Diagnosis x sex × age interaction ASD vs. control χ^2^; p value		1.41; 0.2340	
Diagnosis x sex × age interaction ADHD vs. control χ^2^; p value		1.09; 0.2996	

OR, odds ratio; odds ratio of greater than 1 indicates more adaptive behaviors at older ages, while odds ratio less than one indicates fewer adaptive behaviours at older ages.

When comparing participants with ADHD to controls, the three-way interaction did not reach statistical significance (sex-by-age-by-diagnosis interaction: χ^2^ = 1.25, *p* = 0.3). However, there was a significant sex-by-diagnosis interaction as a result of a strong female advantage in controls compared to females with ADHD across all ages (χ^2^ = 6.04, *p* = 0.01; see **Figure 1**)

The sex-by-age interaction in ASD was also significantly different from the sex-by-age interaction in ADHD (sex-by-age-by-diagnosis: χ^2^ = 24.94, *p* < 0.0001) with better performance in older children than younger children among females with ADHD, but the opposite effect in ASD, where older females performed more poorly than younger females (**Figure 1**).

#### Communication Skill Area

Older children demonstrated higher communication performance than younger children among males with ASD (male OR for age = 1.24, *p* < 0.0001) (**Figure 2**), male controls (male OR for age = 1.96 *p* < 0.0001) (**Figure 2**), and males and females with ADHD (male OR for age = 1.41, *p* < 0.0001; female OR for age = 1.24, *p* = 0.002), with no significant age-by-sex effect in ADHD (χ^2^ = 2.57, *p* = 0.1). In female controls, there was no significant effect of age (female OR for age 2.63, *p* = 0.5), possibly due to ceiling effects occurring after age 9. Similarly, there was no effect of age in females with ASD (female OR for age = 1.03, *p* = 0.6), but there was a significant sex-by-age interaction (χ^2^ = 8.07, *p* = 0.005). No significant age-by-sex effect emerged in controls (χ^2^ = 0.56, *p* = 0.5) (**Figure 2**).

Sex-by-age patterns and the main effects of sex (χ^2^ = 0.003, *p* > 0.9) (χ^2^ = 0.31, *p* = 0.6) were similar between ASD and ADHD (**Figure 3**; **Table 3**); we thus combined these groups for further analyses. When ASD and ADHD were combined into one model, there were significant sex-by-age effects across the pooled sample (χ^2^ = 11.22, *p* = 0.0008). Specifically, females had significantly better scores than males at younger ages (i.e., age 8, OR = 1.27, 95% CI = 1.02-1.58 *p* = 0.03) whereas males had significantly better scores than females at older ages (i.e., age 12, OR = 0.67, 95% CI = 0.51–0.89 *p* = 0.005) (**Figure 3**). Sex-by-age effects (χ^2^ = 1.61, *p* = 0.4) and main effects of sex (χ^2^ = 2.05, *p* = 0.4) for this combined ASD+ADHD group were not significantly different than controls in the communication skill area (**Figure 3**).

#### Leisure Skill Area

Older children obtained higher leisure scores than younger children among males with ASD (male OR for age = 1.13, *p* < 0.0001) (**Figure 4**), female controls (female OR for age = 1.43 *p* = 0.006), and both males and females with ADHD (male OR for age = 1.19, *p* < 0.0001; female OR for age = 1.25, *p* = 0.0002) (**Figure 4**), with no significant age-by-sex effect in ADHD (χ^2^ = 0.58, *p* = 0.5). There was no age effect in male controls (male OR for age = 0.98, *p* = 0.8) or in females with ASD (female OR for age = 0.934, *p* = 0.2) resulting in significant sex-by-age effects in controls (χ^2^ = 6.35, *p* = 0.01) and ASD (χ^2^ = 8.97, *p* = 0.003). Notably, these age-by-sex interactions were in opposite directions across groups, yielding a significant three-way interaction (age-by-sex-by-diagnosis) characterized by better performance with age in females for the control group, but poorer performance with age in females with ASD (χ^2^ = 12.29, *p* = 0.0005) (**Figure 4**). In ADHD, males and females both had better performance with age, but males consistently scored more poorly than females on leisure skills at all age points (χ^2^ = 0.58, *p* = 0.5). Notably, although females and males in both groups had better skills with age, the sex differences with age increased more in controls than those with ADHD (sex-by-age-by-diagnosis interaction: χ^2^ = 3.93, *p* = 0.05) (**Figure 4**).

The sex-by-age interactions were significantly different between ASD and ADHD (sex-by-age-by-diagnosis interaction: χ^2^ = 6.91, *p* = 0.0086) with better scores at older ages than younger ages in females with ADHD but poorer scores at older ages for females with ASD (**Figure 4**).

### Exploratory Analyses

Trait scores from the SWAN, SCQ, and RBSR were highly significant predictors of communication, leisure, and social adaptive abilities (all OR’s < 1, *p’*s < 0.0001) with higher trait scores associated with significantly lower ABAS total scores, across diagnostic groups.

In the combined sample of all participants with neurodevelopmental conditions, previously reported significant diagnosis by sex-by-age interactions remained significant after controlling for SCQ, SWAN, and RBSR (see **Supplementary Table 1**). However, within ASD, a previously reported significant sex-by-age interaction in the communication domain was no longer significant after controlling for SCQ (χ^2^ = 2.78, *p* = 0.1), with a trend noted also in the leisure domain (original sex-by-age interaction in the leisure domain was χ^2^ = 8.98, *p* = 0.003 and after controlling for SCQ, sex-by-age interaction was χ^2^ = 5.10, *p* = 0.02) (**Supplementary Table 2**). Please see **Supplementary Table 3** to see the influence of SCQ, SWAN and RBSR on sex differences in ADHD for social skill area. For instance as seen in **Supplemental Figures 1**–**6**, when RBSR was added to the models, sex differences were virtually unchanged with lines representing the log (odds ratio) for sex overlapping those without RBSR in the model. However, we noted changes in the log (odds ratio) after controlling for SCQ in both ASD and ADHD (**Supplemental Figures 1**–**6**) with the largest changes noted for ASD communication domain. Changes to the log (odds ratio) when adding SWAN to the model were generally smaller than those seen when SCQ was added to the model, with minimal changes to the sex effect.

## Discussion

This study is the first to our knowledge to examine sex differences in social adaptive function across ASD, ADHD, and typically developing controls. Controls outperformed (i.e., higher expected proportion of adaptive behaviors present) both ADHD and ASD groups, with ASD males and females performing worse on adaptive function in all three skill areas. We found that social adaptive function was better or stable across age points in all but the girls with ASD, whose social performance was significantly poorer at the older time points when compared to the younger time points. When compared to males with ASD, females with ASD had poorer function at older ages, despite better performance at younger ages. Sex differences in children with ASD and ADHD were similar to each other in the communication skill area, with females having significantly better scores than males at younger ages, while males had significantly better scores than females at older ages. In the leisure area, both females and males with ADHD had higher scores at older compared to younger ages with females having better scores compared to males across all ages. Finally, exploratory analyses revealed that the severity of the social deficit in children with ASD partially accounted for sex differences in performance on the ABAS-II communication, and potentially leisure skill areas.

The present findings suggest a different trajectory for social adaptive function in females than males with ASD. Our findings are consistent with the only longitudinal study to date to examine sex differences in social abilities in ASD (49). In this study, females with ASD showed less impairment in early social behaviors using the ADI-R (i.e., social imitative, play, seeking, and offering comfort) than males, but greater social impairments (i.e., poor friendships) in adolescence and adulthood. Holtmann et al. (48) and Lord et al. (70) also found social difficulties in adolescent females compared to males. These findings suggest that social deficits may start to emerge for girls when social situations become more complex and when social pressures increase in adolescence, as girls may rely more on communication and interpersonal skills compared to males (49), a conceptualization consistent with the DSM-5 articulation of social deficits as social demands exceeding capacity. Another possibility is that there was a cohort effect, wherein the 8-year-old girls had access to better social skills training programs than the 12-year-old girls early in their development. It also remains possible that other symptoms (e.g., anxiety) may have started to interfere with social function in older girls, but these were not examined in the current study. This issue emphasizes the need for qualitative and quantitative research that examines male and female social and communication functions in multiple contexts and diverse/complex situations over time with typically developing peers, to determine unique challenges that females with ASD experience over time. Of note, there were no significant sex differences in ASD in communication, leisure, or social skill performance when collapsed across age, which is consistent with previous studies that found no significant sex differences in social and communication abilities in children within the age ranges 7–12 (43, 44, 47). This highlights the critical importance of examining age effects when exploring diagnostic group differences in behavioral and functional domains across neurodevelopmental conditions. The current findings regarding ASD were not consistent with some previous literature that reported that adolescent females had fewer social difficulties than males (38, 40–42). However, these studies included mostly older adolescents and adults, did not examine age effects, and included smaller samples.

Our findings for the ADHD group were in line with the current hypothesis and were consistent with some past research that showed more social-communication problems in children with ADHD compared to controls (30, 71). The findings are also consistent with some exiting literature suggesting more peer problems in males relative to females using parent-reported measures (51, 52). However, other studies have reported that females with ADHD were more likely than males to be reported by teachers as being rejected by peers (53, 72) while others found no differences (24, 73). Discrepancies possibly stemmed from use of diverse array of measures and constructs, as well as informants in addition to potential true differences in behavior across settings. Furthermore, some studies either recruited children with no formal diagnosis of ADHD who reported symptoms consistent with ADHD (72, 73) or had children diagnosed with ADHD using the DSM-III criteria (24, 53) and as such may have included a somewhat different population than later studies.

Some limitations of this study are as follows: (1) We may have been underpowered for some comparisons as only 25% of the sample were females, and we had a relatively small typically-developing control group. (2) In addition, parent-reported measures were used to assess social adaptive function, which may be influenced by parental biases and expectations. More importantly, parent reports may miss a lot of nuances in their child’s lived experiences. Additional assessments using structured clinical interviews and observational measures would have been desirable to provide a richer understanding of the symptoms and behaviors of children in the sample. Moreover, self-reported measures for older children may be beneficial in understanding the unique needs and perspectives of older males and females. Our method of dichotomizing the item scores may have mitigated some of the variability due to parental expectation of appropriate frequency of skills. However, we acknowledge that this results in some loss of the variability that would be available by examining the full range of item scores. Both strategies have strengths and limitations, and we acknowledge the limitation. (3) The present study did not control for IQ, but we do note that IQ differences between males and females in the present study were not significant (see **Table 3.1**). (4) Most importantly, this study employed a cross-sectional design and does not account for potential heterogeneity in trajectories. We acknowledge that our findings are limited by the cross-sectional nature of this study. A longitudinal design is required to confirm our findings and determine both the onset of symptom manifestation differences between males and females, as well as individual trajectories over time (45). (5) Finally, we recognize that the dichotomy between ASD and ADHD is not as definitive as suggested (particularly given the co-occurrence of ADHD and ASD in the current sample), but this dichotomy was necessary for group comparison purposes.

### Clinical Implications

Our study highlights the importance of considering potential sex differences in social adaptive function within and across neurodevelopmental disorders. Understanding such differences will ultimately be critical in both improving the diagnostic/prognostic process, and accounting for variability in presentations in males and females (74). A potential implication of the present findings pertains to treatment planning. The particular pattern observed in females with ASD suggests a female-specific trajectory in social communication, that may imply that social interventions may be needed earlier than might be expected given their apparent competence early on, or potentially that different social interventions may be appropriate for females, although our data does not speak to that. Furthermore, the present study provides a foundation upon which future studies can be built. There is an urgent need for longitudinal studies examining sex differences over time in social adaptive function, given the considerable heterogeneity in this population.

## Conclusion

This study examined sex differences in social and communication functions in children with ASD and ADHD compared to typically developing children. Our findings confirm social adaptive function deficits in both ASD and ADHD, with both male and female children with ADHD showing improvements with age, whereas females with ASD had poorer function at older ages, despite an early advantage. Findings will enhance our understanding of sex differences in social adaptive function across disorders, both informing our understanding of underlying biology and in identifying/addressing unique needs for males and females with developmental disorders.

## Data Availability

All datasets analyzed for this study are included in the manuscript and supplementary files. Also, all data will be available through a public domain release in the last quarter of 2019.

## Ethics Statement

This study was reviewed and approved by the Holland Bloorview Kids Rehabilitation Hospital Research Ethics Board. Written and informed parental consent was obtained for all participants under the age of 16.

## Author Contributions

TM contributed to conceptualization, did data analysis, and is primarily responsible for manuscript preparation. AD contributed to the data analysis and manuscript. JB participated in the design, of the study, co-supervised data analytic approaches, and revised and edited manuscript. EA supervised all procedures in this study and manuscript. XL, EK, SG, RN, JC and RS made substantial contributions to the conception, acquisition of the data for the work, and revised and edited the manuscript. All authors have read and approved the final manuscript.

## Funding

This study was funded by the Ontario Brain Institute – Province of Ontario Neurodevelopmental Disorders (POND) Network (grant number: IDP-PND-2018). TM was supported by an Ontario Graduate Scholarship.

## Conflict of Interest Statement

The authors declare that this study received funding from Ontario Brain Institute, an independent non-profit corporation, funded partially by the Ontario government. The opinions, results and conclusions are those of the authors and no endorsement by the Ontario Brain Institute is intended or should be inferred. TM was also supported by an Ontario Graduate Scholarship. EA has served as a consultant to Roche, has received grant funding from Sanofi Canada and SynapDx, has received royalties from APPI and Springer, and received kind support from AMO Pharmaceuticals, honoraria from Wiley, and honorarium from Simons Foundations. RN has received research grants from Roche. RS has received stocks from ehave and research grants from DNA Genotek, Canadian Institutes of Health Research, and Ontario Brain Institute. The above funders played no role in the study design or data collection and analysis, the decision to publish, or preparation of the manuscript. T, JB, AD, XL, EK, SG and JC declare that the research was conducted in the absence of any commercial or financial relationships that could be construed as a potential conflict of interest.

## References

[1] American Psychiatric Association Diagnostic and statistical manual of mental disorders (2013). 5th ed Washington, DC Author. 10.1176/appi.books.9780890425596

[2] Chronis AM Chacko A Fabian GA Wymbs BT Pelham WE Enhancements to the behavioral parent training paradigm for families of children with ADHD: review and future directions. Clin Child Fam Psychol Rev (2004) 7(1) :1–27. 10.1023/B:CCFP.0000020190.60808.a4 15119686

[3] National Autism Spectrum Disorder Surveillance (NASS 2018). Autism spectrum disorder among children and youth in Canada 2018. A report of the National Autism Spectrum Disorder Surveillance System. Retrieved from https://www.canada.ca/en/public-health/services/publications/diseases-conditions/autism-spectrum-disorder-children-youth-canada-2018.html.

[4] Centers for Disease Control and Prevention (CDC) Prevalence of autism spectrum disorder among children aged 8 years—autism and developmental disabilities monitoring network, 11 Sites-United States, 2014 (2018, April 27). Retrieved from: https://www.cdc.gov/mmwr/volumes/67/ss/ss6706a1.htm. 10.15585/mmwr.mm6745a7 PMC5919599 29701730

[5] Williamson D Johnston C Gender differences in adults with attention-deficit/hyperactivity disorder. Clin Psychol Rev (2015) 40:15–27. 10.1016/j.cpr.2015.05.005 26046624

[6] Ames CS White SJ Are ADHD traits dissociable from the autistic profile? Links between cognition and behaviour. J Autism Dev Disord (2011) 41:357– 363. 10.1007/s10803-010-1049-0 20585847

[7] Leyfer OT Folstein SE Bacalman S Davis NO Dinh E Morgan J Comorbid psychiatric disorders in children with autism: interview development and rates of disorders. J Autism Dev Disord (2006) 36 (7):849–861. 10.1007/s10803-006-0123-0 16845581

[8] Ronald A Simonoff E Kuntsi J Asherson P Plomin R Evidence for overlapping genetic influences on autistic and ADHD behaviours in a community twin sample. J Child Psychol Psychiatry Disc (2008) 49:535–542. 10.1111/j.1469-7610.2007.01857.x 18221348

[9] Demopoulos C Hopkins J Davis A A comparison of social cognitive profiles in children with autism spectrum disorders and attention-deficit/hyperactivity disorder: a matter of quantitative but not qualitative difference? J Autism Dev Disord (2012) 43(5) :1157–1170. 10.1007/s10803-012-1657-y 23015110

[10] Grzadzinski R Di Martino A Brady E Mairena MA O’Neal M Petkova E Examining autistic traits in children with ADHD: does the autism spectrum extend to ADHD? J Autism Dev Disord (2011) 41(9):1178–1191. 10.1007/s10803-010-1135-3 21108041 PMC3123401

[11] Kern JK Geier DA Sykes LK Geier MR Deth RC Are ASD and ADHD a continuum? A comparison of pathophysiological similarities between the disorders. J Atten Disord (2015) 19(9):805–827. 10.1177/1087054712459886 23074304

[12] van der Meer JM Oerlemans AM van Steijn DJ Lappenshcar MG de Sonneville LM Buitelaar JK Are autism spectrum disorder and attention-deficit/hyperactivity disorder different manifestations of one overarching disorder? Cognitive and symptom evidence from a clinical and population-based sample. J Am Acad Child Adolesc Psychiatry (2012) 51 (11):1160–1172. e1163. 10.1016/j.jaac.2012.08.024 23101742

[13] Zandt F Prior M Kyrios M Repetitive behaviour in children with high functioning autism and obsessive compulsive disorder. J Autism Dev Disord (2007) 37:251–259. 10.1007/s10803-006-0158-2 16865546

[14] Biederman J Mick E Faraone SV Braaten E Doyle A Spencer T Influence of gender on attention deficit hyperactivity disorder in children referred to a psychiatric clinic. Am J Psychiatr (2002) 159:36–42. 10.1176/appi.ajp.159.1.36 11772687

[15] Fombonne E Epidemiological surveys of autism and other pervasive developmental disorders: an update. J Autism Dev Disord (2003) 33(4):365–387. 10.1023/A:1025054610557 12959416

[16] Fombonne E Epidemiology of pervasive developmental disorders. Pediatr Res (2009) 65(6):591–598. 10.1203/PDR.0b013e31819e7203 19218885

[17] Baird G Simonoff E Pickles A Chandler S Loucas T Meldrum D Prevalence of disorders of the autism spectrum in a population cohort of children in South Thames: the Special Needs and Autism Project (SNAP). Lancet (2006) 368(9531):210–215. 10.1016/S0140-6736(06)69041-7 16844490

[18] Nicholas JS Charles JM Carpenter LA King LB Jenner W Spratt EG Prevalence and characteristics of children with autism-spectrum disorders. Ann Epidemiol (2008) 18(2):130–136. 10.1016/j.annepidem.2007.10.013 18083540

[19] Hartley SL Sikora DM Which DSM-IV-TR criteria best differentiate high-functioning autism spectrum disorder from ADHD and anxiety disorders in older children? Autism (2009) 13(5):485–509. 10.1177/1362361309335717 19759063 PMC3035910

[20] Hattier MA Matson JL Tureck K Horovitz M The effects of gender and age on repetitive and/or restricted behaviors and interests in adults with autism spectrum disorders and intellectual disability. Res Dev Dis (2011) 32(6):2346–2351. 10.1016/j.ridd.2011.07.028 21824745

[21] Mandy W Chilvers R Chowdhury U Salter G Seigal A Skuse D Sex differences in autism spectrum disorder: evidence from a large sample of children and adolescents. J Autism Dev Disord (2012) 42(7):1304–1313. 10.1007/s10803-011-1356-0 21947663

[22] Arnett AB Pennington BF Willcutt EG DeFries JC Olson RK Sex differences in ADHD symptom severity. J Child Psychol Psychiatry (2015) 56:632–639. 10.1111/jcpp.12337 25283790 PMC4385512

[23] Greene RW Biederman J Faraone SV Sienna M Garcia-Jetton J Adolescent outcome of boys with attention-deficit/hyperactivity disorder and social disability: results from a 4-year longitudinal follow-up study. J Consult Clin Psychol (1997) 65:758–767. 10.1037//0022-006X.65.5.758 9337495

[24] Greene RW Biederman J Faraone SV Monuteaux M Mick E DuPre EP Social impairment in girls with ADHD: patterns, gender comparisons, and correlates. J Am Acad Child Adolesc Psychiatry (2001) 40:704–710. 10.1097/00004583-200106000-00016 11392349

[25] Hoza B Mrug S Gerdes AC Hinshaw SP Bukowski WM Gold JA What aspects of peer relationships are impaired in children with attention- deficit/hyperactivity disorder? J Consult Clin Psychol (2005) 73:411–423. 10.1037/0022-006X.73.3.411 15982139

[26] Abikoff H Jensen P Arnold LE Hoza B Hechtman L Pollack S Observed classroom behavior of children with ADHD: relationship to gender and comorbidity. J Abnorm Child Psychol (2002) 30:349–359 10.1023/A:1015713807297 12109488

[27] Blachman DR Hinshaw SP Patterns of friendship among girls with and without attention-deficit/hyperactivity disorder. J Abnorm Child Psychol (2002) 30:625–640. 12481976 10.1023/a:1020815814973

[28] Bickett L Milich R First impressions formed of boys with learning disabilities and attention deficit disorder. J Learn Disabil (1990) 23:253–259. 10.1177/002221949002300409 2324638

[29] Erhardt D Hinshaw SP Initial sociometric impressions of attention-deficit hyperactivity disorder and comparison boys: predictions from social behaviors and from nonbehavioral variables. J Consult Clin Psychol (1994) 62:833–842. 10.1037//0022-006X.62.4.833 7962888

[30] Kok FM Groen Y Fuermaier ABM Tucha O Problematic peer functioning in girls with ADHD: a systematic literature review. PLoS One (2016) 11(11):e0165119. 10.1371/journal.pone.0165119 27870862 PMC5117588

[31] Martel MM Roberts B Gremillion M von Eye A Nigg JT External validation of bifactor model of ADHD: explaining heterogeneity in psychiatric comorbidity, cognitive control, and personality trait profiles within DSM-IV ADHD. J Abnorm Child Psychol (2011) 39(8):1111–1123. 10.1007/s10802-011-9538-y 21735050 PMC3199328

[32] Clark T Feehan C Tinline C Vostanis P Autistic symptoms in children with attention deficit-hyperactivity disorder. Eur Child Adolesc Psychiatry (1999) 8(1):50–55. 10.1007/s007870050083 10367741

[33] Oncu B Oner O Oner P Erol N Aysev A Canat S Symptoms define by parents’ and teachers’ ratings in attention-deficit hyperactivity disorder: changes with age. Can J Psychiatry (2004) 49 (7):487–491. 10.1177/070674370404900711 15362254

[34] Sukhodolsky DG Rosario-Campos MC Scahill L Katsovich L Pauls DL Peterson BS Adaptive, emotional and family functioning of children with obsessive-compulsive disorder and comorbid attention deficit hyperactivity disorder. Am J Psychiatr (2005) 162 (6):1125–1132. 10.1176/appi.ajp.162.6.1125 15930061 PMC2291297

[35] Tye C Asherson P Ashwood KL Azadi B Bolton P McLoughlin G Attention and inhibition in children with ASD, ADHD and co-morbid ASD + ADHD: an event-related potential study. Psychol Med (2014) 44(5):1101–1116. 10.1017/S0033291713001049 23673307

[36] Mikami AY Lorenzi J Gender and conduct problems predict peer functioning among children with attention-deficit/hyperactivity disorder. J Clin Child Adolesc Psychol (2011) 40(5):777–786. 10.1080/15374416.2011.597089 21916696 PMC3174783

[37] Van Wijngaarden-Cremers PJ Eten EV Groen WB Van Deurzen PA Oosterling IJ Van der Gaat RJ Gender and age differences in the core triad of impairments in autism spectrum disorders: a systematic review and meta-analysis. J Autism Dev Disord (2014) 44(3):627–635. 10.1007/s10803-013-1913-9 23989936

[38] Baron-Cohen S The extreme male brain theory of autism. Trends Cogn Sci (2002) 6(6):248–254. 10.1016/S1364-6613(02)01904-6 12039606

[39] Frazier TW Georgiades S Bishop SL Hardan AY Behavioral and cognitive characteristics of females and males with autism in the simons simplex collection. J Am Acad Child Adolesc Psychiatry (2014) 53:329–340. e1–3. 10.1016/j.jaac.2013.12.004 24565360 PMC3935179

[40] Head AM McGillivray JA Stokes MA Gender differences in emotionality and sociability in children with autism spectrum disorders. Mol Autism (2014) 5(19):1–19. 10.1186/2040-2392-5-19 24576331 PMC3945617

[41] Lai MC Lombardo MB Ruigrok AN Chakrabarti B Wheelwright SJ Auyeung B Cognition in males and females with autism: similarities and differences. PLoS One (2012) 7(10):e47198. 10.1371/journal.pone.0047198 23094036 PMC3474800

[42] Sedgewick F Hill V Yates R Pickering L Pellicano E Gender differences in the social motivation and friendship experiences of autistic and non-autistic adolescents. J Autism Dev Disord (2015) 46(4):1297–1306. 10.1007/s10803-015-2669-1 PMC4786616 26695137

[43] May T Cornish K Rinehart NJ Gender profiles of behavioral attention in children with autism spectrum disorder. J Atten Disord (2016) 20:627–35. 10.1177/1087054712455502 22912506

[44] Park S Cho SC Cho IH Kim BN Kim JW Shin MS Sex differences in children with autism spectrum disorders compared with their unaffected siblings and typically developing children. Res Autism Spectr Disord (2012) 6(2):861–870. 10.1016/j.rasd.2011.11.006

[45] Rivet TT Matson JL Review of gender differences in core symptomatology in autism spectrum disorders. Res Autism Spectr Disord (2011) 23(3):957–976. 10.1016/j.rasd.2010.12.003

[46] Sipes M Matson JL Worley JA Kozlowski AM Gender differences in symptoms of autism spectrum disorders in toddlers. Res Autism Spectr Disord (2011) 5(4):1465–1470. 10.1016/j.rasd.2011.02.007

[47] Solomon M Miller M Taylor SL Hinshaw SP Carter CS Autism symptoms and internalizing psychopathology in girls and boys with autism spectrum disorders. J Autism Dev Disord (2012) 42(1):48–59. 10.1007/s10803-011-1215-z 21442362 PMC3244604

[48] Holtmann M Bölte S Poustka F Autism spectrum disorders: sex differences in autistic behaviour domains and coexisting psychopathology—ProQuest. Dev Med Child Neurol (2007) 49(5):361–366. 10.1111/j.1469-8749.2007.00361.x 17489810

[49] McLennan JD Lord C Schopler E Sex differences in higher functioning people with autism. J Autism Dev Disord (1993) 23:217–227. 10.1007/BF01046216 8331044

[50] Thurber JR Heller TL Hinshaw SP The social behaviours and peer expectations of girls with attention deficit hyperactivity disorder and comparison girls. J Clin Child Adolesc Psychol (2002) 31(4):443–452. 10.1207/153744202320802124 12402564

[51] Thorell LB Rydell A-M Behavior problems and social competence deficits associated with symptoms of attention-deficit hyperactivity disorder: effects of age and gender. Child Care Health Dev (2008) 34:584–595. 10.1111/j.1365-2214.2008.00869.x 18796051

[52] Pelham WE Bender ME Peer relationships in hyperactive children: description and treatment. In: Gadow KD Bailer I, editors. Advances in learning and behavioral disabilities. (1982) vol. 1 JAI Press . p. 365–436.

[53] Berry CA Shaywitz SE Shaywitz BA Girls with attention deficit disorder: a silent majority? A report on behavioral and cognitive characteristics. Pediatrics (1985) 76:801–809. 4058990

[54] Veenstra R Lindenberg S Oldehinkel AJ De Winter AF Verhulst FC Ormel J Prosocial and antisocial behavior in preadolescence: teachers’ and parents’ perceptions of the behavior of girls and boys. Int J Behav Dev (2008) 32(3):243–251. 10.1177/0165025408089274

[55] Hall JA Carter JD Horgan TG Gender differences in nonverbal communication of emotion. In: Fischer AH, editor. Gender and emotion: Social psychological perspectives (2000). Cambridge University Press p. 97–117. 10.1017/CBO9780511628191.006

[56] Walker S Gender differences in the relationship between young children’s peer-related social competence and individual differences in theory of mind. J Genet Psychol (2005) 2005(166):297–312. 10.3200/GNTP.166.3.297-312 16173673

[57] Barbu S Cabanes G Le Maner-Idrissi G Boys and girls on the playground: sex differences in social development are not stable across early childhood. PLoS One (2011) 6(1):e16407. 10.1371/journal.pone.0016407 21297987 PMC3030576

[58] Rose AJ Rudolph KD A review of sex differences in peer relationship processes: potential trade-offs for the emotional and behavioral development of girls and boys. Psychol Bull (2006) 132:98–131. 10.1037/0033-2909.132.1.98 16435959 PMC3160171

[59] Larson R Richards MH Introduction: the changing life space of early adolescence. J Youth Adolesc (1989) 18:501–509. 10.1007/BF02139070 24272122

[60] Lord C Cook EH Leventhal BL Amaral DG Autism spectrum disorder. Neuron (2000) 28:355–363. 10.1016/S0896-6273(00)00115-X 11144346

[61] Lord C Rutter M Le Couteur A Autism diagnostic interview-revised: a revised version of a diagnostic interview for caregivers of individuals with possible pervasive developmental disorders. J Autism Dev Disord (1994) 24:659–685. 10.1007/BF02172145 7814313

[62] Ickowicz A Schachar RJ Sugarman R Chen SX Millette C Cook L The parent interview for child symptoms: a situation-specific clinical research interview for attention-deficit hyperactivity and related disorders. Can J Psychiatry (2006) 51:325–328. 10.1177/070674370605100508 16986822

[63] Harrison P Oakland T Adaptive Behavior Assessment System (2003). 2nd ed San Antonio, TX: Psychological Corporation.

[64] Wechsler D Wechsler Abbreviated Scale of Intelligence (1999). San Antonio, TX: Psychological Corporation. 10.1037/t15170-000

[65] Wechsler D Wechsler Abbreviated Scale of Intelligence—Second Edition (2011). San Antonio, TX: Psychological Corporation. 10.1037/t15171-000

[66] Wechsler D Wechsler Intelligence Scale for Children—Fourth Edition (2003). San Antonio, TX: Psychological Corporation. 10.1037/t15174-000

[67] Thorndike R Hagen EP Saltier JM Stanford-binet intelligence scale. 4th ed Chicago, IL: Riverside (1986).

[68] Rutter M Bailey A Lord C The Social Communication Questionnaire: Manual. Western Psychological Services (2003).

[69] Swanson JM Schuck S Porter MM Carlson C Hartman CA Sergeant JA Categorical and dimensional definitions and evaluations of symptoms of ADHD: history of the SNAP and SWAN rating scales. TIJEPA (2012) 10(1):51. PMC4618695 26504617

[70] Lord C Schopler E Revecki D Sex differences in autism. J Autism Dev Disord (1982) 12:317–330. 10.1007/BF01538320 7161234

[71] Hinshaw SP Preadolescent girls with attention-deficit/hyperactivity disorder: I. Background characteristics, comorbidity, cognitive and social functioning, and parenting practices. J Consult Clin Psychol (2002) 70:1086–1098. 10.1037//0022-006X.70.5.1086 12362959

[72] Diamantopoulou S Henricsson L Rydell AM ADHD symptoms and peer relations of children in a community sample: examining associated problems, self-perceptions, and gender differences. Int J Behav Dev (2005) 29(5):388–398. 10.1080/01650250500172756

[73] DeHaas P Attention styles and peer relationships of hyperactive and normal boys and girls. J Abnorm Child Psychol (1986) 14:457–467. 10.1007/BF00915438 3760350

[74] Kreiser NL White SW ASD in females: are we over-stating the gender difference in diagnosis? Clin Child Fam Psychol Rev (2014) 17(1):67–84. 10.1007/s10567-013-0148-9 23836119

