# Supplementary material for: Sex Differences in Social Adaptive Function in Autism Spectrum Disorder and Attention-Deficit Hyperactivity Disorder
Source: Front Psychiatry. 2019 Sep 12;10:607. doi: 10.3389/fpsyt.2019.00607 (PMC6751776; doi:10.3389/fpsyt.2019.00607)
Supplement: Supplementary file 1 [file DataSheet_1.docx]

Testing whether SCQ, SWAN and RBSR influences sex differences across disorder for each skill area by comparing sex by age by diagnosis interactions before and after controlling for individual trait measure.

**Supplemental Table 1:** Influence of SCQ, SWAN and RBSR on sex differences across disorder for each skill area

|  | Sex by Age by Dx interaction | Sex by Age by Dx After Controlling for SCQ | | Sex by Age by Dx After Controlling for SWAN | | Sex by Age by Dx After Controlling for RBSR | Overall  Outcome |
| --- | --- | --- | --- | --- | --- | --- | --- |
| Leisure skill area | **Significant** (Chi-square=6.91, p=0.009) | **Significant** (Chi-square=6.42, p=0.001) | **Significant** (Chi-square=6.83, p=0.009) | | **Significant** (Chi-square=9.61, p=0.002) | | Trait measures have little influence |
| Social skill area | **Significant** (Chi-square=24.93, p<0.0001) | **Significant** (Chi-square=23.11, p<0.0001) | **Significant** (Chi-square=24.84, p<0.0001) | | **Significant** (Chi-square=30.35, p>0.0001) | | Trait measures have little influence |

**Supplemental Table 2:** Influence of SCQ, SWAN and RBSR on sex differences in ASD for each skill area

Testing whether SCQ, SWAN and RBSR influences sex differences in ASD for each skill area by comparing sex by age interactions before and after controlling for individual trait measure.

| ASD | Sex by Age Interaction | Sex by Age Interaction after controlling for SCQ | | Sex by Age Interaction after controlling for SWAN | | Sex by Age Interaction after controlling for RBSR | Overall Outcome |
| --- | --- | --- | --- | --- | --- | --- | --- |
| Communication skill area | Significant (Chi-square=8.07, p=0.005) | **Not Significant (Chi-square=2.78, p=0.1)** | Significant (Chi-square=8.91, p=0.0028) | | Significant (Chi-square=10.80, p=0.0010) | | Sex differences in ASD on the communication domain can be accounted for by differences in male and female scores on the SCQ |
| Leisure skill area | Significant (Chi-square=8.98, p=0.003) | Significant (Chi-square=5.10, p=0.02) | Significant (Chi-square=9.39, p=0.002) | | Significant (Chi-square=11.19, p=0.0008) | | Trait measures have little influence |
| Social skill area | Significant (Chi-square=26.03, p<0.0001) | Significant (Chi-square=17.49, p<0.0001) | Significant (Chi-square=27.71, p<0.0001) | | Significant (Chi-square=30.60, p<0.0001) | | Trait measures have little influence |

**Supplemental Table 3:** Influence of SCQ, SWAN and RBSR on sex differences in ADHD for social skill area

Testing whether SCQ, SWAN and RBSR influences sex differences in ADHD for social skill area by comparing sex by age interactions before and after controlling for individual trait measure.

| ADHD | Sex by Age Interaction | Sex by Age Interaction after controlling for SCQ | | Sex by Age Interaction after controlling for SWAN | | Sex by Age Interaction after controlling for RBSR | Overall Outcome |
| --- | --- | --- | --- | --- | --- | --- | --- |
| Social skill area | Significant (Chi-square=5.47, p=0.01) | Significant (Chi-square=7.78, p=0.005) | Significant (Chi-square=4.62, p=0.032) | | Significant (Chi-square=7.08, p=0.008) | | Trait measures have little influence |


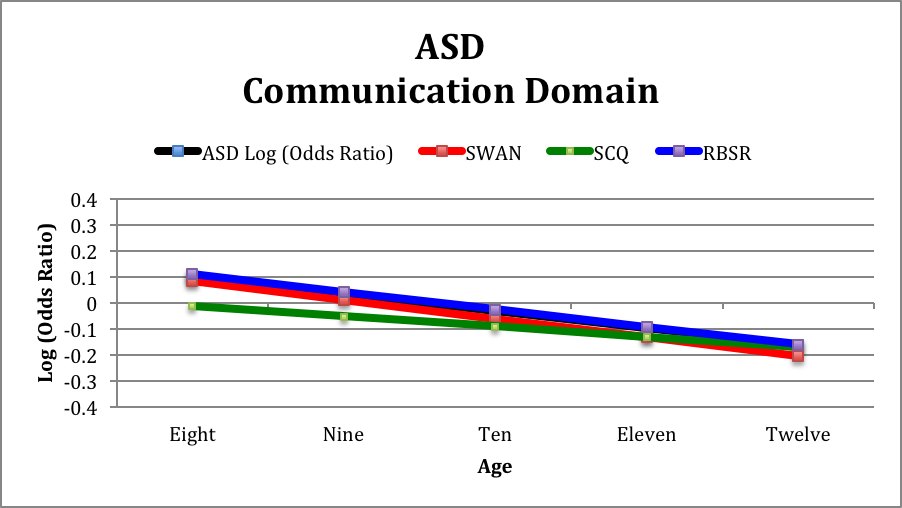


Graph is the log of the odds of females obtaining a positive score of 1 over the odds of males obtaining a positive score of 1 across all ages. A log odds ratio of above 0 indicates a higher odds for females with ASD to obtain a positive score of 1 in the communication domain, while a log odds ratio of below 0 indicates higher odds for males with ASD to obtain a positive score of 1 in the communication domain. The black line indicates the log of odds ratio before controlling for the trait measures (i.e. SCQ, SWAN and RBSR). The red line represents the log odds ratio after controlling for SWAN, while the green line indicates the log odds ratio after controlling for SCQ, and the blue line indicates the log odds ratio after controlling for RBSR.

**Supplemental Figure 1:** Log (Odds Ratio) by Age: ASD Communication

Graph is the log of the odds of females obtaining a positive score of 1 over the odds of males obtaining a positive score of 1 across all ages. A log odds ratio of above 0 indicates a higher odds for females with ADHD to obtain a positive score of 1 in the communication domain, while a log odds ratio of below 0 indicates higher odds for males with ADHD to obtain a positive score of 1 in the communication domain. The black line indicates the log of odds ratio before controlling for the trait measures (ie. SCQ, SWAN and RBSR). The red line represents the log odds ratio after controlling for SWAN, while the green line indicates the log odds ratio after controlling for SCQ, and the blue line indicates the log odds ratio after controlling for RBSR.

**Supplemental Figure 2:** Log (Odds Ratio) by Age: ADHD Communication


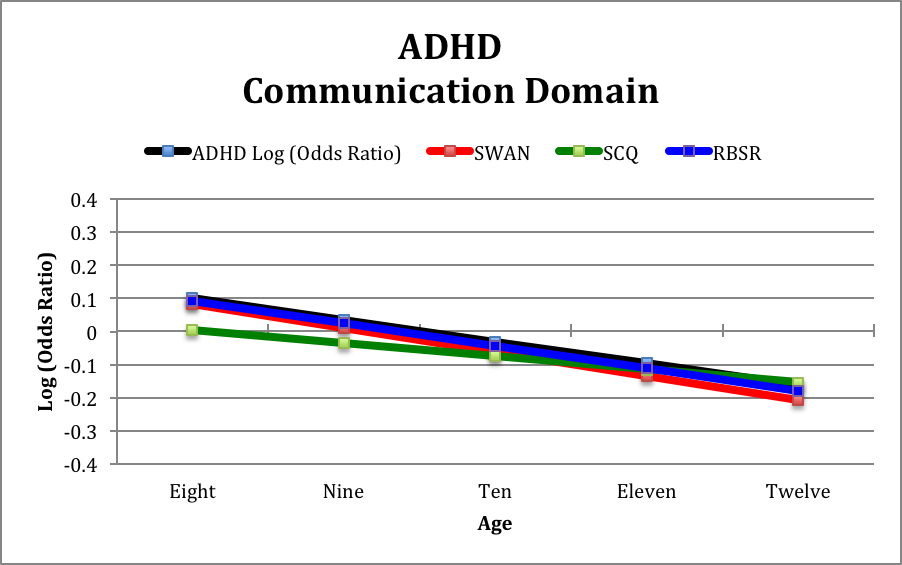


Graph is the log of the odds of females obtaining a positive score of 1 over the odds of males obtaining a positive score of 1 across all ages. A log odds ratio of above 0 indicates a higher odds for females with ASD to obtain a positive score of 1 in the leisure domain, while a log odds ratio of below 0 indicates higher odds for males with ASD to obtain a positive score of 1 in the leisure domain. The black line indicates the log of odds ratio before controlling for the trait measures (ie. SCQ, SWAN and RBSR). The red line represents the log odds ratio after controlling for SWAN, while the green line indicates the log odds ratio after controlling for SCQ, and the blue line indicates the log odds ratio after controlling for RBSR.


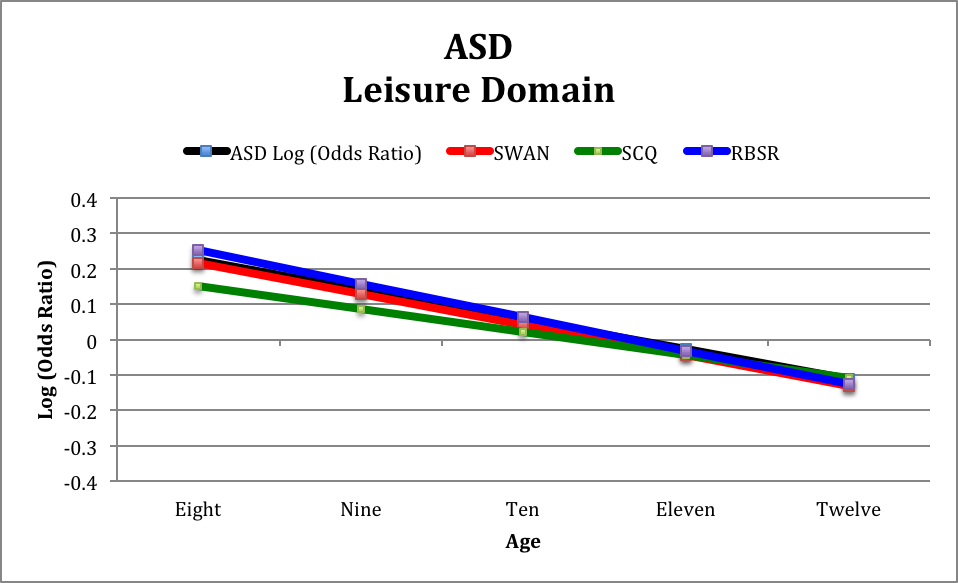


**Supplemental Figure 3:** Log (Odds Ratio) by Age: ASD Leisure


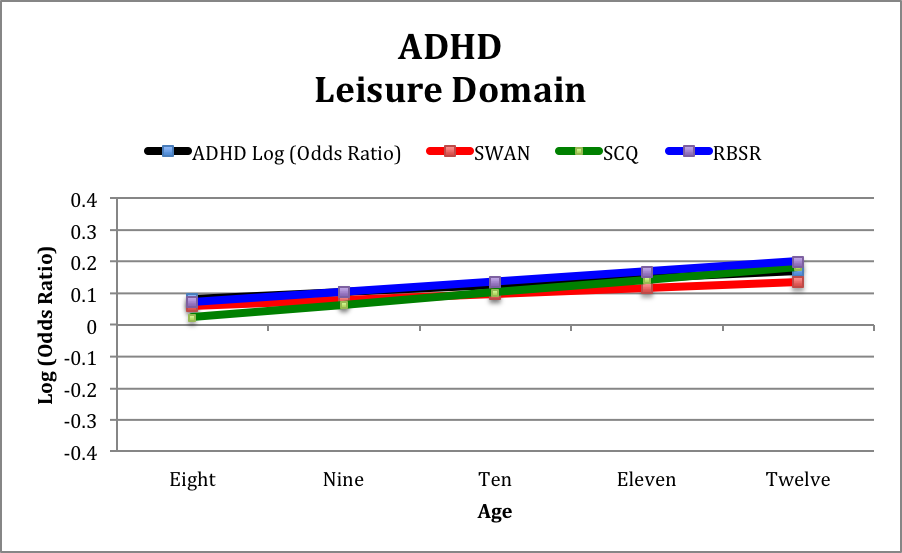


Graph is the log of the odds of females obtaining a positive score of 1 over the odds of males obtaining a positive score of 1 across all ages. A log odds ratio of above 0 indicates a higher odds for females with ADHD to obtain a positive score of 1 in the leisure domain, while a log odds ratio of below 0 indicates higher odds for males with ADHD to obtain a positive score of 1 in the leisure domain. The black line indicates the log of odds ratio before controlling for the trait measures (ie. SCQ, SWAN and RBSR). The red line indicates the log odds ratio after controlling for SWAN, while the green line indicates the log odds ratio after controlling for SCQ, and the blue line indicates the log odds ratio after controlling for RBSR.

**Supplemental Figure 4:** Log (Odds Ratio) by Age: ADHD Leisure

Graph is the log of the odds of females obtaining a positive score of 1 over the odds of males obtaining a positive score of 1 across all ages. A log odds ratio of above 0 indicates a higher odds for females with ASD to obtain a positive score of 1 in the social domain, while a log odds ratio of below 0 indicates higher odds for males with ASD to obtain a positive score of 1 in the social domain. The black line indicates the log of odds ratio before controlling for the trait measures (ie. SCQ, SWAN and RBSR). The red line indicates the log odds ratio after controlling for SWAN, while the green line indicates the log odds ratio after controlling for SCQ, and the blue line indicates the log odds ratio after controlling for RBSR.

**Supplemental Figure 5:** Log (Odds Ratio) by Age: ASD Social


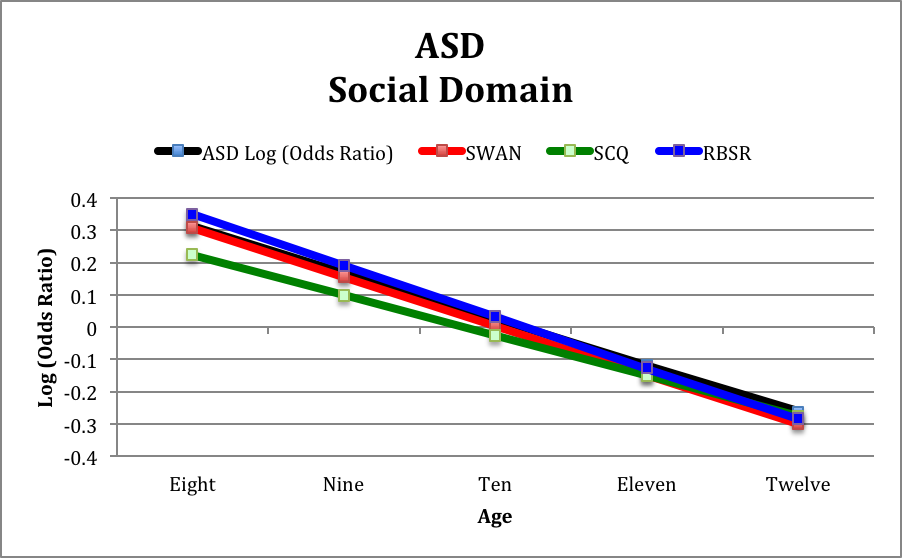


**Supplemental Figure 6:** Log (Odds Ratio) by Age: ADHD Social

Graph is the log of the odds of females obtaining a positive score of 1 over the odds of males obtaining a positive score of 1 across all ages. A log odds ratio of above 0 indicates a higher odds for females with ADHD to obtain a positive score of 1 in the social domain, while a log odds ratio of below 0 indicates higher odds for males with ADHD to obtain a positive score of 1 in the social domain. The black line indicates the log of odds ratio before controlling for the trait measures (ie. SCQ, SWAN and RBSR). The red line indicates the log odds ratio after controlling for SWAN, while the green line indicates the log odds ratio after controlling for SCQ, and the blue line indicates the log odds ratio after controlling for RBSR.


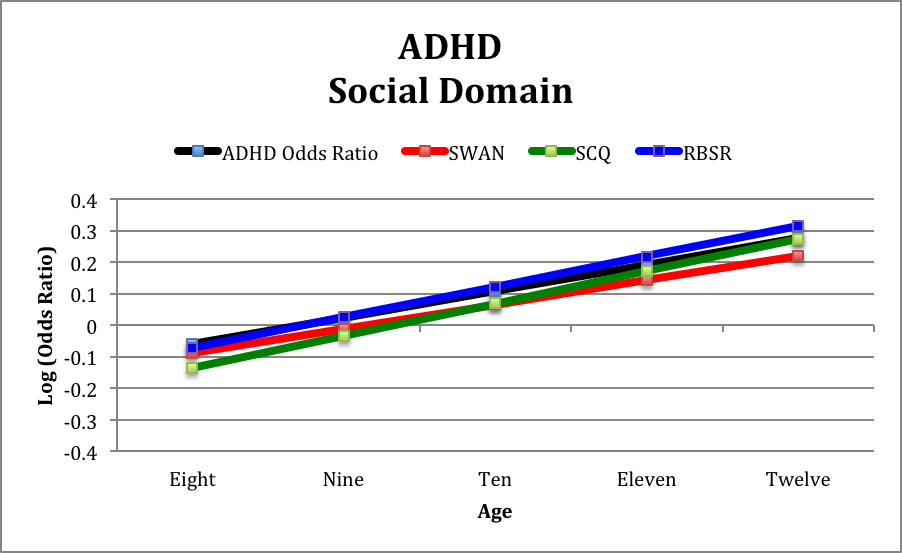


| ASD | Seven | | Eight | | Nine | | Ten | | Eleven | | Twelve | |
| --- | --- | --- | --- | --- | --- | --- | --- | --- | --- | --- | --- | --- |
|  | Male | Female | Male | Female | Male | Female | Male | Female | Male | Female | Male | Female |
| IQ | 83.5 (20.6) | 81.0 (4.2) | 77.8 (23.9) | 99.0 (23.0) | 78.8 (23.8) | 78.5 (20.5) | 91.2 (21.1) | 83.7 (11.9) | 90.6 (27.4) | 82.6 (10.2) | 82.3 (28.4) | 91.3 (37.0) |
| ABAS Communication | 15.0 (4.3) | 17.0 (2.8) | 17.4 (5.8) | 17.8 (5.7) | 15.2 (6.3) | 14.5 (3.5) | 18.4 (5.3) | 16.1 (5.8) | 19.7 (4.4) | 18.4 (4.9) | 20.1 (3.7) | 19.0 (5.2) |
| ABAS Leisure | 14.3 (4.2) | 14.0 (1.4) | 13.5 (5.2) | 16.5 (3.0) | 12.3 (5.1) | 15.5 (4.9) | 14.1 (4.8) | 13.9 (6.7) | 16.2 (3.5) | 16.2 (3.8) | 17.4 (2.8) | 14.5 (2.6) |
| ABAS Social | 15.5 (5.6) | 18.0 (5.7) | 15.2 (6.1) | 18.0 (6.4) | 11.4 (5.7) | 16.5 (6.4) | 16.8 (5.9) | 13.0 (4.4) | 16.6 (5.2) | 16.8 (3.0) | 20.1 (2.4) | 15.3 (6.7) |
| SCQ | 19.4 (9.8) | 18.3 (10.4) | 20.1 (6.8) | 16.7 (9.9) | 23.4 (4.8) | 18.0 (7.1) | 20.6 (6.4) | 20.7 (8.1) | 19.3 (6.5) | 18.7 (5.1) | 16.5 (8.4) | 18.3 (11.3) |
| SWAN | 9.1 (5.7) | 7.0 (8.5) | 7.1 (4.4) | 9.0 (6.0) | 9.9 (5.8) | 9.5 (12.0) | 8.5 (5.8) | 9.3 (0.7) | 9.0 (4.0) | 4.6 (4.6) | 7.5 (5.5) | 7.4 (3.7) |
| RBSR | 8.5 (6.9) | 21.0 (19.8) | 11.8 (8.8) | 8.5 (8.4) | 12.8 (11.3) | 5.0 (5.7) | 7.7 (6.6) | 12.7 (3.5) | 7.2 (6.5) | 6.4 (4.1) | 8.6 (9.8) | 7.4 (10.5) |

**Supplemental Table 4**- Mean Scores (SD) by Age for ASD: Table presents mean scores on IQ, ABAS communication, leisure and social total scores, SCQ, SWAN and RBSR for children with ASD ages 8, 9, 10, 11 and 12.

| ADHD | Seven | | Eight | | Nine | | Ten | | Eleven | | Twelve | |
| --- | --- | --- | --- | --- | --- | --- | --- | --- | --- | --- | --- | --- |
|  | Male | Female | Male | Female | Male | Female | Male | Female | Male | Female | Male | Female |
| IQ | 102.2 (15.0) | 98.5 (14.1) | 102.2 (18.8) | 90.7 (10.7) | 100.5 (16.7) | 91.3 (25.8) | 105.5 (11.9) | 98.0 (20.3) | 102.9 (19.1) | 105.3 (6.9) | 103.2 (16.9) | 124.0  (-) |
| ABAS Communication | 19.9 (2.3) | 19.4 (4.1) | 19.5 (4.1) | 22.3 (2.1) | 21.1 (3.2) | 20.8 (3.0) | 22.7 (2.3) | 21.6 (2.4) | 23.1 (1.5) | 22.7 (1.0) | 22.9 (1.7) | 22.0  (-) |
| ABAS Leisure | 15.9 (2.8) | 15.5 (4.5) | 16.8 (3.1) | 18.8 (2.6) | 17.7 (3.1) | 17.5 (2.9) | 18.6 (3.3) | 19.4 (2.5) | 18.7 (2.6) | 18.6 (3.2) | 18.4 (2.7) | 20.0  (-) |
| ABAS Social | 19.8 (3.1) | 18.9 (4.0) | 20.9 (2.8) | 20.6 (1.8) | 20.1 (3.2) | 21.0 (3.4) | 20.6 (3.2) | 21.0 (2.7) | 20.8 (1.8) | 21.6 (2.2) | 21.3 (2.0) | 23.0  (-) |
| SCQ | 8.2 (5.5) | 8.7  (6.8) | 7.0 (5.0) | 5.4 (4.1) | 8.8 (6.4) | 6.5  (5.4) | 8.0 (4.6) | 7.0 (5.5) | 5.9 (5.0) | 5.5 (5.7) | 6.2 (5.2) | 9.0  (-) |
| SWAN | 11.4 (5.3) | 11.0 (5.4) | 8.1 (4.4) | 9.4 (5.1) | 11.1 (4.6) | 3.5 (4.0) | 14.0 (3.1) | 9.2 (4.6) | 9.4 (4.8) | 11.0 (4.5) | 8.6 (5.2) | 10.0  (-) |
| RBSR | 5.0 (6.2) | 3.3 (4.0) | 3.4 (5.8) | 4.8 (8.0) | 3.3 (4.1) | 2.0 (2.8) | 4.0 (5.3) | 3.5 (5.4) | 2.1 (2.7) | 5.3 (4.4) | 1.5 (1.8) | 0.0  (-) |

**Supplemental Table 5**-Mean Scores (SD) by Age for ADHD: Table presents mean scores on IQ, ABAS communication, leisure and social total scores, SCQ, SWAN and RBSR for children with ADHD ages 8, 9, 10, 11 and 12.

| Controls | Seven | | Eight | | Nine | | Ten | | Eleven | | Twelve | |
| --- | --- | --- | --- | --- | --- | --- | --- | --- | --- | --- | --- | --- |
|  | Male | Female | Male | Female | Male | Female | Male | Female | Male | Female | Male | Female |
| IQ | 114.6 (8.1) | 115.5 (6.0) | 107.9 (8.9) | 111.0 (20.8) | 106.4 (10.7) | 113.8 (11.0) | 105.3 (17.8) | 117.0 (10.8) | 115.5 (12.5) | 110.5 (3.5) | 106.0 (4.4) | 109.5 (9.2) |
| ABAS Communication | 22.2 (1.8) | 22.3 (1.7) | 22.6 (1.6) | 24.0 (0.0) | 24.0 (0.0) | 24.0 (0.0) | 23.6 (0.8) | 24.0 (0.0) | 23.9 (0.4) | 24.0 (0.0) | 24.0 (0.0) | 23.8 (0.5) |
| ABAS Leisure | 20.4 (0.9) | 19.8 (1.3) | 19.1 (2.5) | 20.3 (1.2) | 21.0 (1.0) | 21.0 (0.0) | 20.7 (1.2) | 21.8 (0.5) | 19.4 (2.6) | 21.5 (0.7) | 19.3 (2.6) | 21.3 (1.5) |
| ABAS Social | 22.0 (1.0) | 22.3 (1.5) | 21.7 (1.9) | 23.0 (0.0) | 23.0 (0.0) | 23.0 (0.0) | 22.8 (0.4) | 22.8 (0.5) | 22.3 (1.1) | 23.0 (0.0) | 21.5 (1.7) | 23.0 (0.0) |
| SCQ | 3.5 (1.8) | 1.0 (1.0) | 4.7 (3.3) | 2.3 (1.2) | 2.0 (1.4) | 1.6 (1.5) | 1.7 (1.1) | 1.0 (1.2) | 1.7 (1.1) | 3.6 (3.4) | 4.3 (1.8) | 2.3 (1.0) |
| SWAN | 0.0 (0.0) | 0.0 (0.0) | 0.5 (1.0) | 0.0 (0.0) | 0.0 (0.0) | 0.0 (0.0) | 0.1 (0.3) | 0.0 (0.0) | 0.1 (0.3) | 4.0 (5.7) | 0.3 (0.5) | 0.0 (0.0) |
| RBSR | 0.4 (0.9) | 0.0 (0.0) | 0.2 (0.6) | 0.3 (0.6) | 0.0 (0.0) | 0.0 (0.0) | 0.0 (0.0) | 0.3 (0.5) | 0.0 (0.0) | 2.0 (2.8) | 0.0 (0.0) | 0.0 (0.0) |

**Supplemental Table 6**- Mean Scores (SD) by Age for Controls: Table presents mean scores on IQ, ABAS communication, leisure and social total scores, SCQ, SWAN and RBSR for typically developing controls ages 8, 9, 10, 11 and 12.
